# Synthetic vs. non-synthetic sweeteners: their differential effects on gut microbiome diversity and function

**DOI:** 10.3389/fmicb.2025.1531131

**Published:** 2025-05-15

**Authors:** Alex Kidangathazhe, Theresah Amponsah, Abhijit Maji, Seidu Adams, Maria Chettoor, Xiuqing Wang, Joy Scaria

**Affiliations:** ^1^Department of Veterinary and Biomedical Sciences, South Dakota State University, Brookings, SD, United States; ^2^Department of Biology and Microbiology, South Dakota State University, Brookings, SD, United States; ^3^Department of Veterinary Pathobiology, Oklahoma State University, Stillwater, OK, United States

**Keywords:** artificial sweetener, gut microbiome, bioreactor, acesulfame K, rebaudioside A, saccharin, sucralose, xylitol

## Abstract

The rising use of artificial sweeteners, favored for their zero-calorie content and superior sweetness, necessitates understanding their impact on the gut microbiome. This study examines the effects of five common artificial sweeteners—Acesulfame K, Rebaudioside A, Saccharin, Sucralose, and Xylitol—on gut microbiome diversity using minibioreactor arrays. Fecal samples from three healthy individuals were used to inoculate bioreactors that were subsequently supplemented with each sweetener. Over 35 days, microbial diversity and network composition were analyzed. Results revealed synthetic sweeteners like Sucralose and Saccharin significantly reduced microbial diversity, while non-synthetic sweeteners, particularly Rebaudioside A and Xylitol, were less disruptive. Acesulfame K increased diversity but disrupted network structure, suggesting potential long-term negative impacts on microbiome resilience. Sucralose enriched pathogenic families such as Enterobacteriaceae, whereas natural sweeteners promoted beneficial taxa like Lachnospiraceae. Random Matrix Theory (RMT) based analysis highlighted distinct microbial interaction patterns, with Acesulfame K causing persistent structural changes. Findings suggest non-synthetic sweeteners may be more favorable for gut health than synthetic ones, emphasizing cautious use, particularly for those with gut health concerns. This study enhances our understanding of artificial sweeteners’ effects on the gut microbiome, highlighting the need for further research into their long-term health implications.

## Introduction

As larger portions of the population become aware of the adverse effects of overconsuming natural sugar, many now turn towards zero-calorie alternative sweeteners. These alternative sweeteners can be both synthetic or artificial, but all are non-nutritive; that is, they have negligible caloric content yet are usually sweeter than natural sugar (Radenkovic, 2023; Kossiva et al., 2024). They are substituted in place of sugar in various foods, sometimes becoming even more prevalent than normal sugar. Such alternative sweeteners are added to foods such as desserts, sodas, cereals, dairy products, powdered drink mixes, baked goods, candy, chocolates, puddings, canned foods, jams and jellies, and confectionery chewing gums.

All mainstream sweeteners, including acesulfame K, aspartame, neotame, advantame, saccharin, and sucralose (with the exception of cyclamate, which has not yet been approved by the FDA) (Sun and Xu, 2025), have been approved for human consumption, given that the allowable daily intake (ADI) is followed (Feng et al., 2024). Since humans cannot metabolize these sweeteners, there are very few direct effects resulting from their consumption. However, previous literature has shown that although the human digestive system cannot directly interact with such sweeteners, another equally important part of the human body, i.e., gut microbiome can and is impacted by it (Lee et al., 2024; Gauthier et al., 2024; Conz et al., 2023; Angelin et al., 2024).

The human gut microbiome is affected by all things that enter the gut, and because of its importance in maintaining homeostasis, any change in the gut can affect the overall health of the human. Previous literature has shown that there are definite interactions between zero-calorie alternative sugars and the gut microbiome (Liu et al., 2024). These interactions have been studied in rats, mice, piglets, hamsters, and humans themselves (Gauthier et al., 2024; Suez et al., 2022; Cyphert et al., 2024). However, there has been a great disparity in the results of such studies, with different sweeteners producing both positive and negative effects on overall health (Conz et al., 2023; Shil et al., 2024).

In this context, bioreactors offer a simple model system for determining the effect of food additives such as artificial sweeteners. Bioreactors offer several key advantages as a model system for studying the gut microbiome compared to animal models (Auchtung et al., 2015; Auchtung et al., 2016). They allow for precise control and reproducibility of environmental factors, enabling the isolation and study of specific variables. Bioreactors facilitate real-time monitoring and sampling, providing insights into dynamic changes over time. For example, bioreactor model has been used to monitor the real-time dynamics of the gut microbiome in response to antibiotic treatment, revealing the rapid shifts in microbial composition and the subsequent recovery process (Guzman-Rodriguez et al., 2018). This real-time monitoring capability allows for a deeper understanding of the temporal dynamics of the gut microbiome, which is more challenging to achieve in animal models. Moreover, bioreactors are cost-effective, ethically advantageous, and offer flexibility and high throughput compared to animal models. Furthermore, bioreactor systems has the potential as an alternative to animal models for studying the gut microbiome, reducing cost, increasing throughput, and the ability to test multiple conditions simultaneously (Agrinier et al., 2024). The use of bioreactors also reduces the need for animal testing, aligning with the principles of the 3Rs (Replacement, Reduction, and Refinement) in animal research (Russell and Burch, 1959). Additionally, bioreactors can be inoculated with human-derived gut microbiota, increasing the relevance of findings to human health. We have previously used minibioreactor arrays to study the modulation of human gut microbiome by dietary fiber and flavonoids (Ghimire et al., 2021). This human-derived inoculation approach enhances the translatability of bioreactor findings to human gut health and disease.

In this study, using minibioreactors arrays, we analyzed the impact of five major artificial sweeteners, i.e., Acesulfame K, Rebaudioside A, Saccharin, Sucralose, and Xylitol on human gut microbiome. Our results show that although all artificial sweeteners tested caused changes to gut microbial diversity indices and network composition, non-synthetic sweeteners were less disruptive when compared to synthetic sweeteners.

## Methods

### Selection of artificial sweeteners and bioreactor experiments

To ensure that our results would be broadly relevant across human populations, we chose five of the most commonly used artificial sweeteners in food: sucralose, saccharin, rebaudioside A, xylitol, and acesulfame K. We used a minibioreactor array as the model system to determine the impact of these artificial sweeteners on the gut microbiome. Fecal samples were donated by three individuals who had not taken antibiotics in the previous year and did not have any known health issues (e.g., obesity or diarrhea), serving as the source microbiome inoculum in the minibioreactor array. The fecal samples were collected following approval from South Dakota State University Institutional Review Board and were processed in an anaerobic chamber following procedures we published previously (Ghimire et al., 2021). We used modified Brain Heart Infusion Broth (BHI) as it has been shown to support the growth of a broad range of gut bacteria (Fenske et al., 2020; Ghimire et al., 2020). All media and solutions used were pre-reduced in advance. The mini-bioreactors were sterilized, assembled with minor modifications according to previous literature, and placed in a Coy anaerobic chamber maintained at 37°C. Modified BHI media was used as the control, while treatment groups received the sweeteners dissolved in the media. The final experimental groups consisted of modified BHI with the addition of sucralose (96.88 mg/L), acesulfame K (290.6 mg/L), rebaudioside A (232.5 mg/L), xylitol (968.8 mg/L), and saccharin (96.88 mg/L). The input and output of the Watson Marlow pumps were set at 1 and 2 rpm, respectively, and the rotating magnetic stirrer was set at 130 rpm. The media were allowed to flow continuously for 24 h each in 4 replicates. Three hundred microliters of the inoculum was introduced into all replicates with a retention time of 16 h. The minibioreactors were then operated for up to 35 days post-inoculation. The sweeteners were introduced to the media and allowed to run continuously from day 7 to day 21. After treatment with the sweeteners, a run with media lacking artificial sweeteners was continued until the last day of the experiment (day 35). Five hundred microliters of the media was collected for optical density (OD) and pH estimation, as well as sequencing, at days 2, 7, 14, 21, and 35, and directly frozen at −80°C. OD and pH of the samples were estimated using a biophotometer (Eppendorf) and an Orion Star A211 pH meter (Thermo Scientific), respectively.

### Microbial community sequencing and bioinformatics analysis of the data

DNA isolation was performed on 144 samples. The DNA was extracted from 500 μL of each sample using a Powersoil DNA isolation kit (MoBio Laboratories Inc., CA) following the manufacturer’s instructions. After extraction, the quality of DNA was measured using NanoDrop™ One (Thermo Fisher Scientific, DE) and quantified using a Qubit Fluorometer 3.0 (Invitrogen, CA). The DNA samples were stored at −20°C until further use. To analyze the variation in microbial composition over time, all samples were amplicon sequenced using an Illumina MiSeq platform with 300 base paired-end V3 chemistry. The library was prepared using an Illumina Nextera XT library preparation kit (Illumina Inc., CA) targeting the V3-V4 regions of the 16S rRNA gene. The libraries were bead normalized and multiplexed before loading into the sequencer. We used QIIME (Bolyen et al., 2019) for Operational Taxonomic Unit (OTU) assignment and taxonomic classification. DADA2 (Callahan et al., 2016) was used as the classifier with the GreenGenes database for taxonomy (DeSantis et al., 2006), and QIIME was otherwise run with default settings. The resulting table was filtered to remove low-abundance species, which may have been anomalies, and then combined with the taxonomic output. Data was further analyzed using R packages such as superheat for the heatmap. The networks were created by grouping the combined OTU and taxonomic data into periods 7–21 (treatment) and periods 28–35 (withdrawal). For the Random Matrix Theory (RMT) based network analysis of the community, these datasets were then independently imported into the program MENA (Deng et al., 2012), where the networks were created and then exported to Cytoscape (Cline et al., 2007; Smoot et al., 2011). Modularity was calculated using the short random walks methodology, and MENA calculated node degree and node centrality. Cytoscape was used to format and correspond network statistics and the visual layout of the network.

## Results

### Artificial sweeteners induce differential alterations in microbiome diversity and enrich some taxa, particularly members of *Bacillaceae*

**Figure 1:**
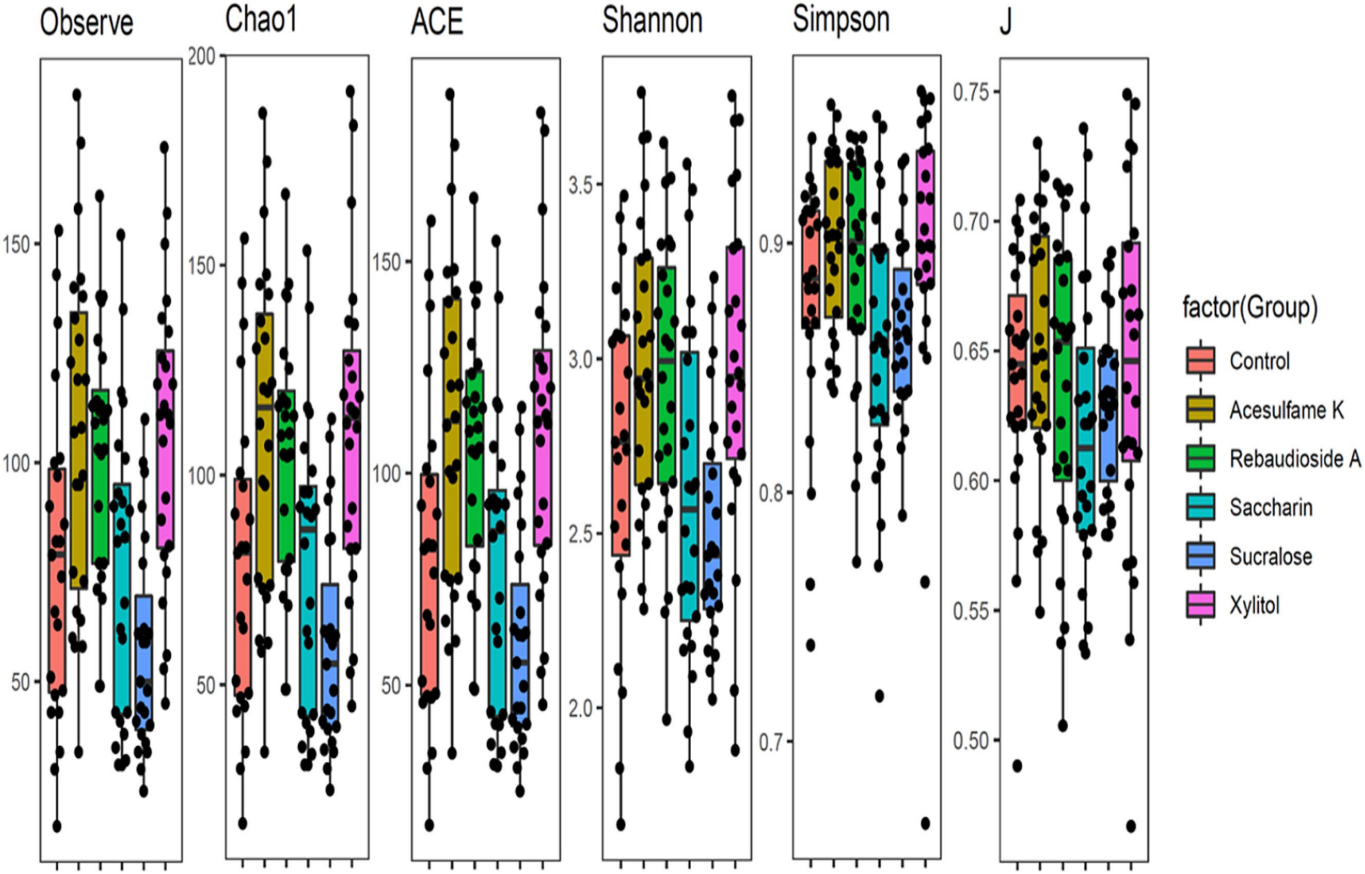
Box plots showing diversity metrics of gut microbiota across different artificial sweetener Treatments- The black dots represent individual data points within each group.

To comprehensively understand the impact of the tested artificial sweeteners on the microbial community in the bioreactors, we calculated multiple diversity indices (Figure 1). All indices showed clear and consistent changes in alpha diversity. Three treatments—acesulfame K, rebaudioside A, and xylitol—appeared to slightly increase diversity across all indices. The Observed, Chao1, and ACE indices clearly indicated that acesulfame K provided the greatest increase in diversity. The Shannon, Simpson, and J indices showed a nearly equal increase from both acesulfame K and xylitol, with rebaudioside A being marginally lower or comparable. In contrast, saccharin and sucralose decreased diversity across all indices. However, while saccharin caused only a marginal decrease, sucralose led to a much larger and more significant reduction in diversity. These results highlight the differential effects of artificial sweeteners on microbial community diversity, with some sweeteners promoting diversity while others have detrimental impacts.

**Figure 2:**
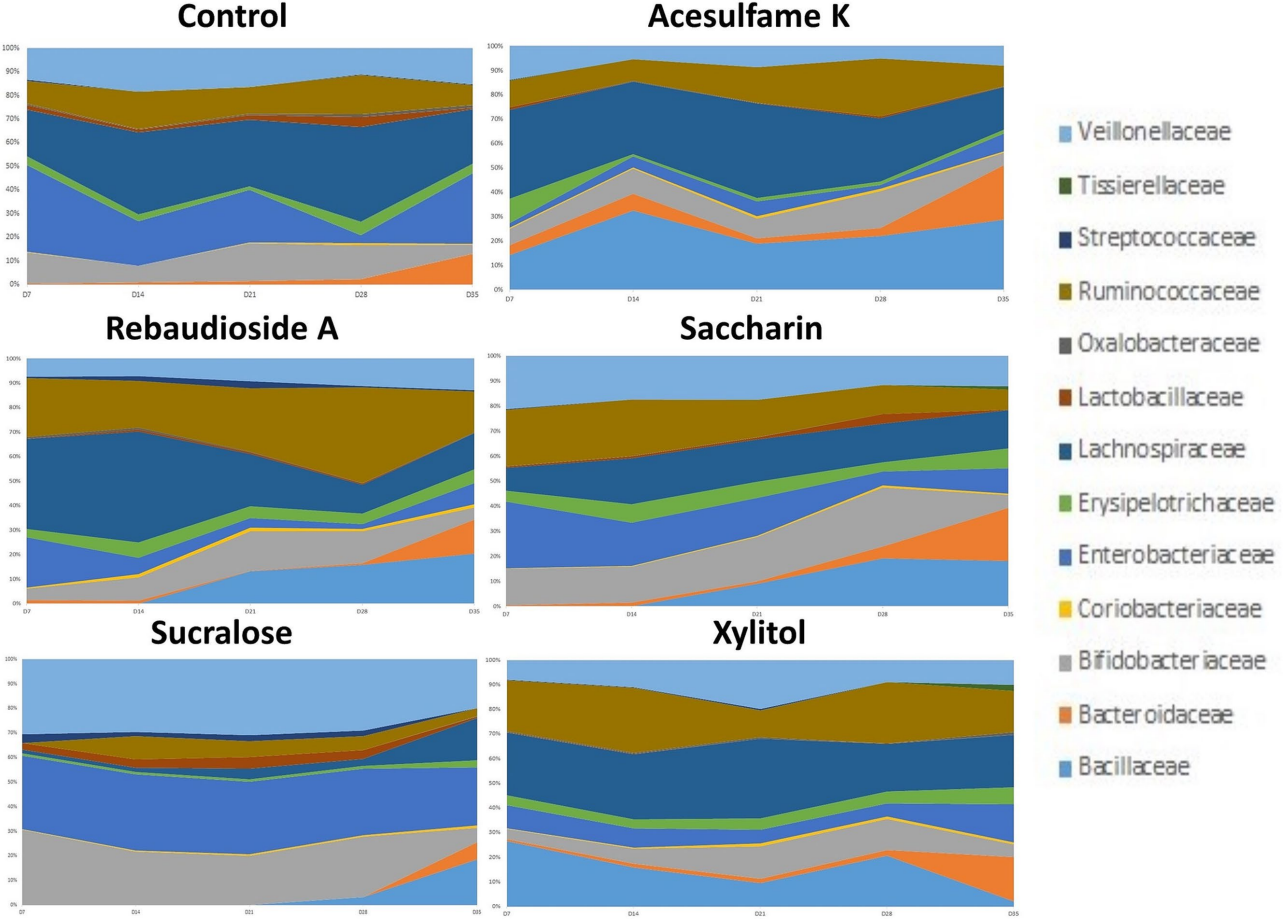
Compositional changes of microbiome at family level. The most abundant 13 bacterial taxa at day 7, 14, 21, 28, and 35 are shown. Days 7–21 are artificial sweetener supplementation period. Days 22–35 are withdrawal period.

Next, we examined the microbiome community compositional changes at the family level. Our experimental approach included a one-week stabilization phase (days 1–7), followed by a two-week artificial sweetener supplementation period (days 7–21) and a 2-week no supplementation phase (days 21–35). The supplementation phase aimed to determine which taxa were enriched or depleted, while the no supplementation phase was intended to assess whether the microbiota could revert to its original composition after the intervention ceased. This analysis (Figure 2) revealed that all treatments exhibited varying levels of change compared to the control. Sucralose was the most divergent from the control, enriching members of *Veillonellaceae*, *Bifidobacteriaceae*, and Enterobacteriaceae while suppressing others to less than 10% of the total community. Interestingly, all sweeteners enriched *Bacillaceae*, although this family was not detected in the control group. Among the sweeteners, acesulfame K consistently showed the strongest enrichment of *Bacillaceae*. After the treatment was withdrawn, all the sweetener treated groups began trending towards a composition similar to the control. However, none of them fully reverted to the original community composition by the end of the 14-day withdrawal period.

**Figure 3:**
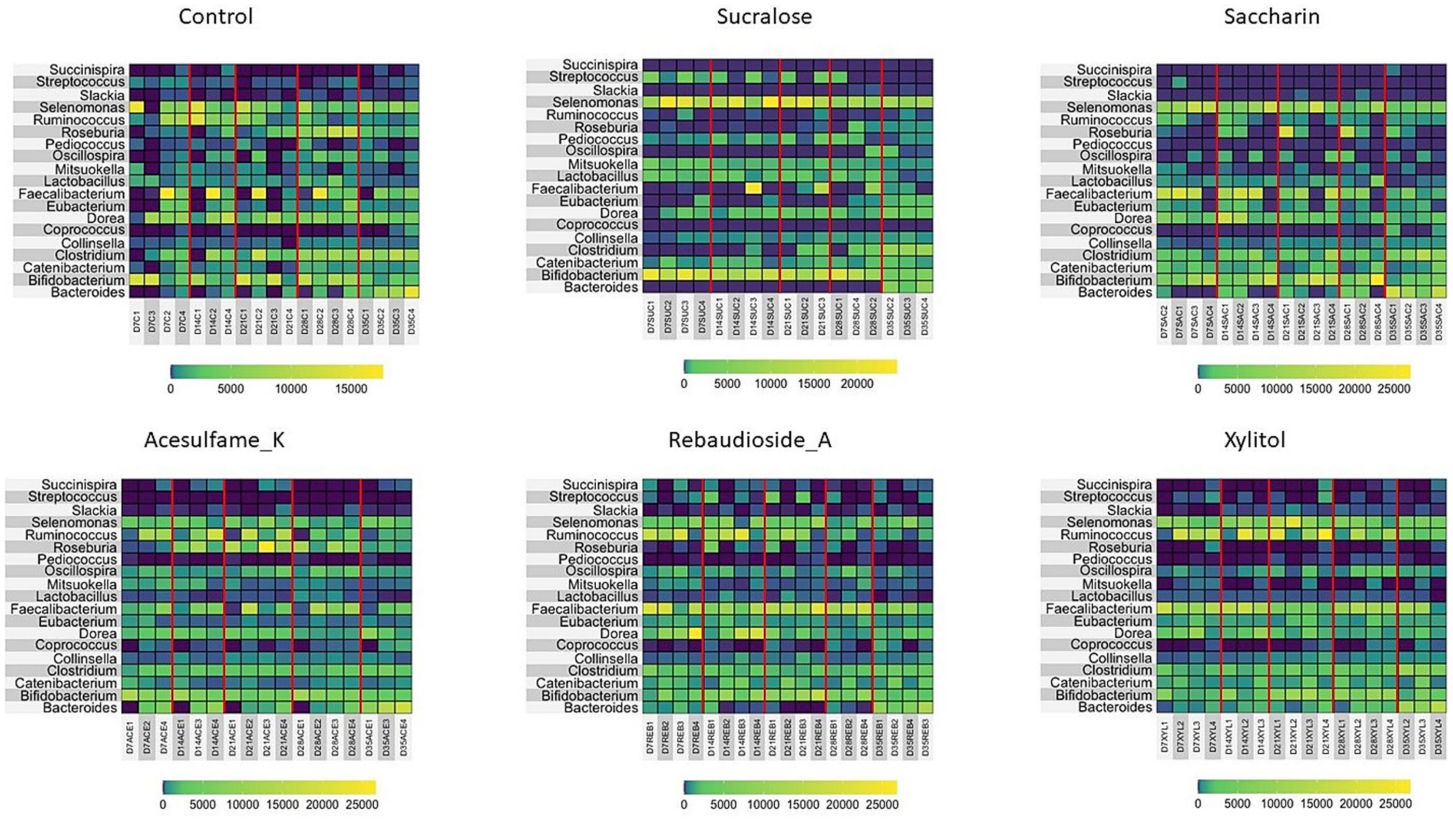
Compositional changes of microbiome at genus level. Heatmap showing the most abundant 13 bacterial taxa at day 7, 14, 21, 28, and 35 are shown. Days 7–21 are artificial sweetener supplementation period. Days 22–35 are withdrawal period.

Motivated by the enrichment of specific families in the treated groups, we further investigated the microbiome composition at the genus level. Figure 3 presents the top 19 genera found in each treatment group. While all treatments enriched multiple genera, sucralose uniquely enriched *Bifidobacterium*, a genus not observed in any other treatment. *Lactobacillus* and *Ruminococcus* were also enriched, consistent with the family-level analysis. *Succinispira* and *Streptococcus* exhibited large variations in abundance depending on the treatment administered; however, rebaudioside A was the only sweetener that enriched both genera. Other notably enriched genera included *Selenomonas* by sucralose and saccharin. Interestingly, *Pediococcus* was eliminated in all treatments except sucralose. These findings underscore the persistent and differential impact of artificial sweeteners on the gut microbiome composition, highlighting the need for further research to understand the long-term consequences of these changes on host health and well-being.

### Random matrix theory (RMT) based network analysis reveals distinct impacts of artificial sweeteners on microbiome community structure and interactions

**Figure 4:**
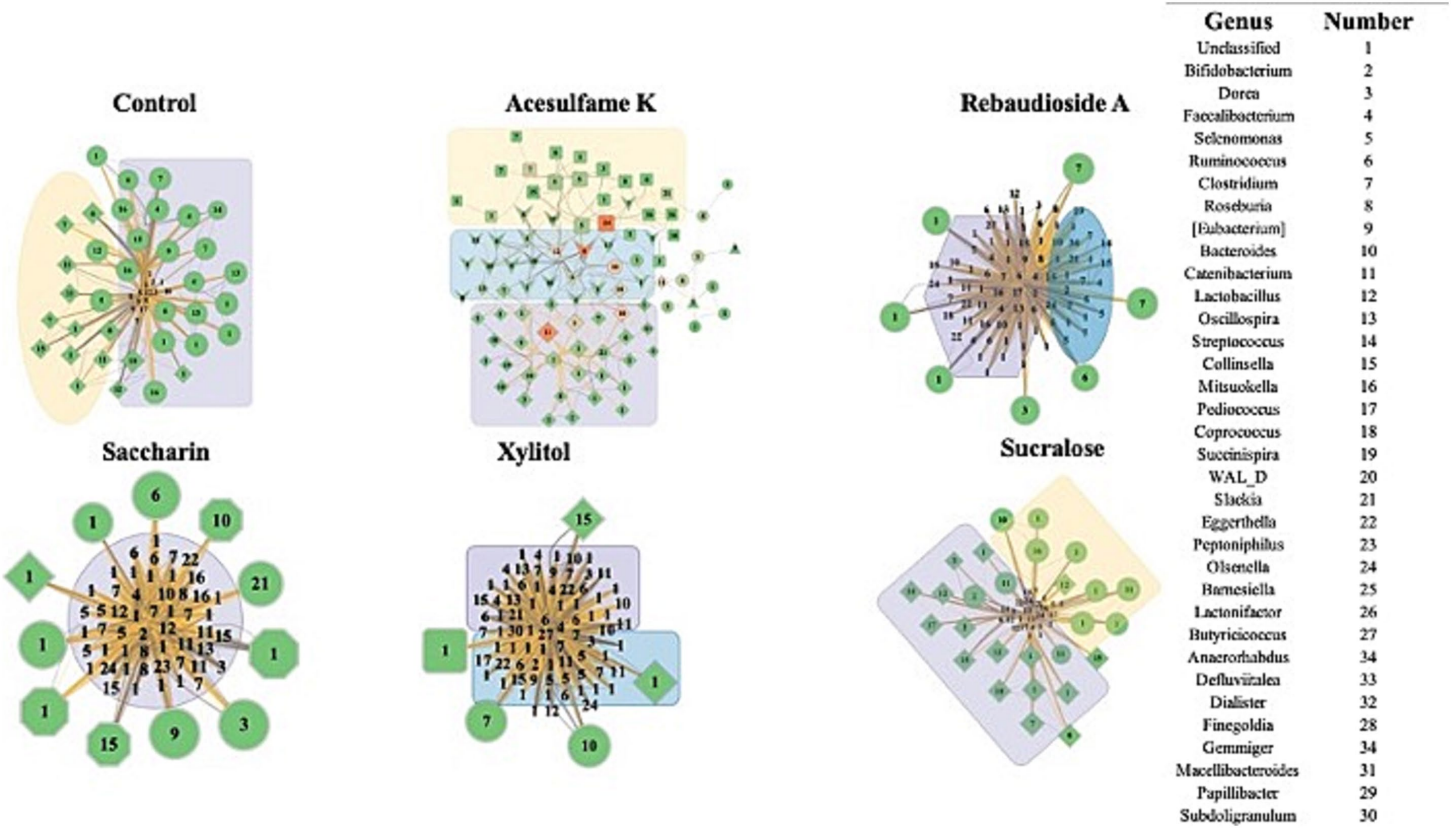
Impact of artificial sweeteners on the community composition during treatment period. RMT based Network created in MENAP with the samples from days 14–21 separated by treatment. Node size is node-degree and node color represents a gradient of node centrality. The edge color represents positive and negative connections between nodes. The shape of the node represents the module it is part of. The colored annotations are the top 3 modules by number of nodes going from Purple (1), Yellow (2), and Blue (3) however there will be no annotation if there are less than 20 nodes in a module.

To gain deeper insights into the impact of the treatments on the microbiome, we employed Random Matrix Theory (RMT) based network analysis of the treated community. RMT analysis is a powerful approach that uncovers complex interactions and relationships among microbial taxa and identifies distinct microbial modules that are influenced by a given treatment. Figure 4 presents the network of the microbial communities during treatment (days 14–21). Structurally, it is evident that even on days 14–21, some stabilization of the bioreactor is yet to occur, as demonstrated by the large imbalance in node degree and centrality in all treatments, excluding acesulfame K. In the case of acesulfame K, although it increases diversity, it significantly alters the overall community structure by reducing its centrality and interconnectedness. Acesulfame K is the only treatment with three modules, and these modules are far less interconnected and centrally located compared to all other treatments. It is also important to note that every species in the acesulfame K treatment possesses the same node degree, indicating that the average number of connections between bacteria is quite low but uniform across the entire community. This suggests that a general island-forming phenomenon has occurred. Regarding the other treatments, most structurally resemble the control, with a significant portion of the bacterial species having extremely low node degrees surrounded by several highly important bacteria. Although this may be entirely due to the lack of full community stabilization, the similarity persists. The impacts on diversity are clearly visible, with sucralose having the fewest number of species and rebaudioside A having a multitude of equally insignificant species. Saccharin and xylitol are intermediate in terms of the quantity of bacterial species and are similar to the control but have a greatly diminished number of key species.

**Figure 5:**
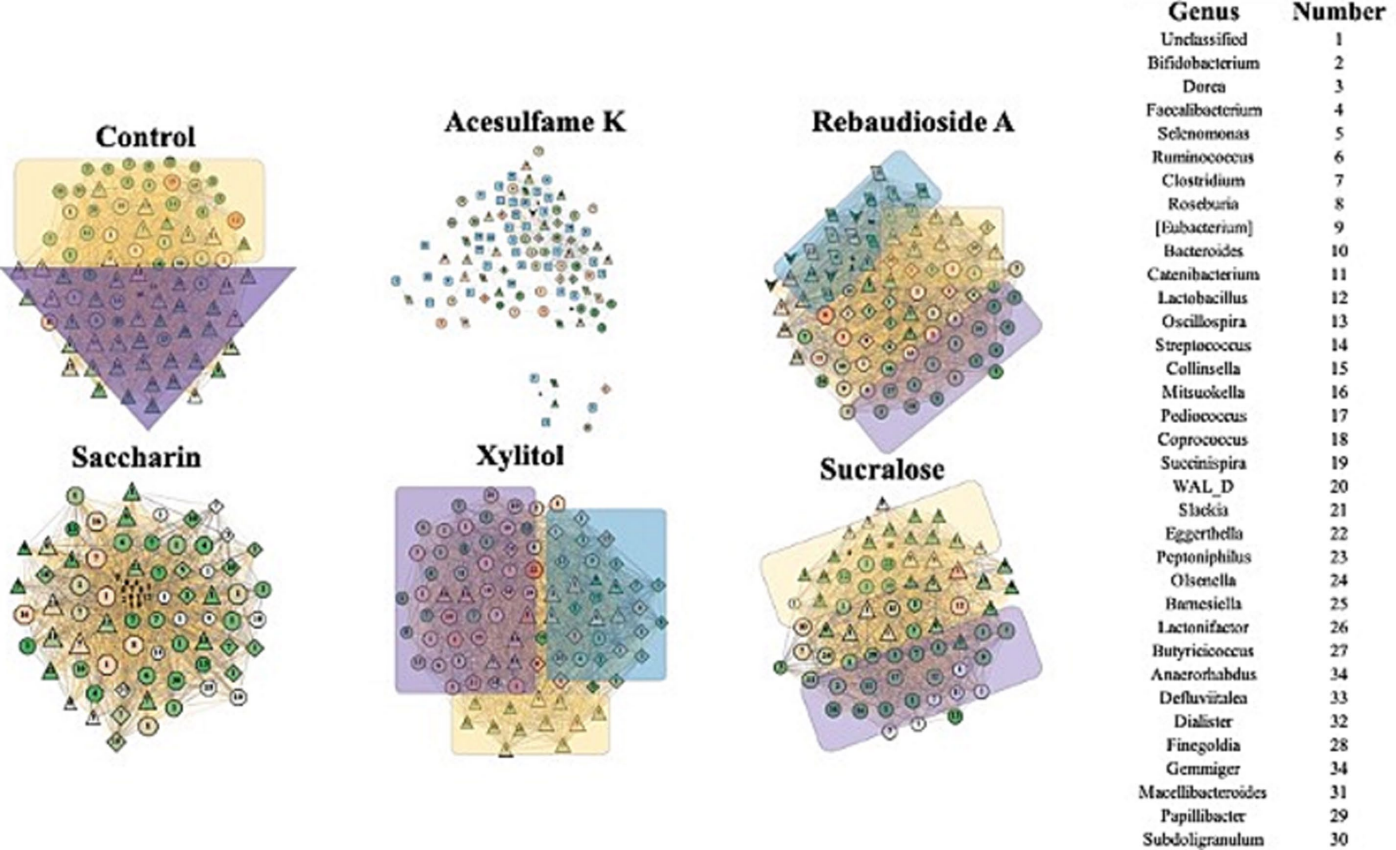
Impact of artificial sweeteners on the community composition during withdrawal period. RMT Network created in MENAP with the samples from days 28–35 separated by treatment. Node size is node-degree and node color is gradient of node-centrality. The edge color represents positive and negative connections between nodes. The shape of the node represents the module it is part of. The colored annotations are the top 3 modules by number of nodes going from Purple (1), Yellow (2), and Blue (3) however there will be no annotation if there are less than 20 nodes in a module.

Figure 5 shows the results of the network analysis during the withdrawal period (days 21–35). The most immediately notable observation is the similarity between all treatments and the control, with the exception of acesulfame K. After being treatment-free for 2 weeks, these treatments exhibit a far more balanced and natural network structure. Acesulfame K is the only treatment that has become less structurally defined. Even after the treatment has been fully withdrawn for 2 weeks, all modularity has disappeared. Furthermore, several completely isolated islands have split from the main network, which has decreased in both centrality and degree. Rebaudioside A, sucralose, and xylitol have all become extremely similar in structure to the control, albeit with fewer nodes in some cases. In the case of saccharin, significant structural recovery has been made; however, several bacteria remain heavily suppressed in the center of the network. Although these bacteria remain connected to the main network, they are in contrast to the isolated islands observed in the acesulfame K treatment. These findings suggest that while most artificial sweeteners allow the microbiome community to recover its structure after the withdrawal period, acesulfame K has a more lasting impact on the network properties and interactions.

## Discussion

When discussing artificial sweeteners and their impact on the gut microbiome, it is crucial to consider their origin and manufacturing process. Two of the sweeteners in the study can be considered natural: rebaudioside A and xylitol. Rebaudioside A is the sweetening component in Stevia (a plant) (Gardana et al., 2003; Peteliuk et al., 2021), whereas xylitol, although found in most plants, is usually produced industrially through chemical means (Umai et al., 2022). It is also important to note that sucralose is a chlorinated sugar substitute, meaning that in its manufacturing process, sucrose has three of its hydroxyl groups replaced with chlorine atoms (Hellwig, 2024). Saccharin and acesulfame K are also fully synthetic but have no notable components that would explain their effects (Wang and Lin, 2025).

While previous research generally suggests that artificial sweeteners negatively impact diversity (Castañeda-Monsalve et al., 2024; Shou et al., 2024), our study finds that acesulfame K, rebaudioside A, and xylitol increased diversity. Rebaudioside A and xylitol are both natural sweeteners, leading us to believe that naturally derived sugar substitutes may be more beneficial for diversity compared to their synthetic counterparts. This aligns with previous studies suggesting that natural sweeteners have less disruptive effects on the gut microbiota (Serrano et al., 2021). We also hypothesize that the significant drop in diversity observed with sucralose could be due to the presence of chlorine in its manufacturing process, as chlorine is antibacterial (Jani, 2019).

Our analysis at the family level shows that while all sweeteners induce change, sucralose induced severe alterations, enriching potentially harmful families like Enterobacteriaceae, which includes pathogenic genera such as Escherichia and Citrobacter. This can be potentially detrimental to human health as these bacteria are often associated with gut inflammation and dysbiosis. In contrast, rebaudioside A and xylitol, which are naturally based, promoted beneficial families such as Lachnospiraceae and Ruminococcaceae, which are important for the production of short-chain fatty acids (SCFAs) like butyrate, essential for gut health. These trends are also observed at the genus level. Although sucralose significantly enriched Bifidobacterium and Lactobacillus, typically considered beneficial (Oerlemans et al., 2021; Xiao et al., 2021; Zoghi et al., 2021; Zacarchenco et al., 2022), this came at the cost of overall diversity, indicating a selective pressure that may not be entirely beneficial in the long term. Rebaudioside A and xylitol promoted the genus Ruminococcus (Ze et al., 2012; Reichardt et al., 2014), which plays a critical role in digesting complex carbohydrates and SCFA production, despite their treatment not having a large prevalence of complex carbohydrates. The presence of SCFAs like butyrate and propionate is crucial for maintaining intestinal barrier integrity and reducing inflammation (Ghimire et al., 2021; Rios-Covian et al., 2015; Seekatz et al., 2018).

RMT-based network analysis was key in understanding how artificial sweeteners structurally alter the community. Acesulfame K stood out in its lack of centrality and modularity, which can lead to a less resilient microbiome, making it more susceptible to external stressors and less capable of recovering from perturbations. It was also the only sweetener that did not begin to trend towards the control structurally after treatment was withdrawn, implying that the structural harm caused by acesulfame K is more pervasive and longer-lasting than that caused by other sweeteners. While all other sweeteners had similar structures after treatment was withdrawn, saccharin still heavily suppressed several species. During treatment, the suppression of a large percentage of the community was observed, suggesting that the community would be more susceptible to external stressors and less capable of recovering from perturbations.

While these findings contribute to our understanding of the complex interactions between artificial sweeteners and the gut microbiome, there are some limitations to our study. First, our results are based on testing fecal microbiota samples from a small number of donors. It is well known that the composition of the human gut microbiota can vary substantially among individuals and across populations (Mancabelli et al., 2024; Joos et al., 2025). Therefore, testing a larger cohort of donor samples will be needed to determine whether our results hold true on a broader population scale. Second, our study examined the effect of five common artificial sweeteners over a relatively short period, which may not capture the potential longer-term effects of these compounds. The onset of conditions such as type 2 diabetes, metabolic syndrome, and obesity—linked to artificial sweetener consumption—often requires prolonged exposure. Many studies have reported a link between artificial sweetener intake and an increased risk of type 2 diabetes mellitus, potentially driven by changes in gut microbiota composition, higher glucose absorption, and insulin insensitivity (Okoro and Markus, 2025; Zhang et al., 2024). Although artificial sweeteners are frequently marketed as weight-management aids, long-term use has been associated in some research with weight gain, metabolic syndrome (a cluster of conditions including high blood pressure and abnormal cholesterol levels), and obesity (Khalil et al., 2024; Jiang et al., 2024). Consequently, our short-term findings may not be fully applicable to these conditions. Finally, the functional consequences of taxa enrichment, such as that of Enterobacteriaceae, need further validation in animal models, for example, in mice.

## Data Availability

Raw sequence data from this study is deposited in NCBI SRA under Bioproject ID: PRJNA1125406.
